# Analysis of Emotion Regulation in Spanish Adolescents: Validation of the Emotion Regulation Questionnaire

**DOI:** 10.3389/fpsyg.2015.01959

**Published:** 2016-01-07

**Authors:** Olga Gómez-Ortiz, Eva M. Romera, Rosario Ortega-Ruiz, Rosario Cabello, Pablo Fernández-Berrocal

**Affiliations:** ^1^Department of Psychology, University of CordobaCórdoba, Spain; ^2^Department of Psychology, Social Work and Counselling, University of GreenwichLondon, UK; ^3^Department of Developmental and Educational Psychology, University of GranadaGranada, Spain; ^4^Department of Basic Psychology, University of MalagaMálaga, Spain

**Keywords:** emotion regulation, reappraisal, suppression, assessment, adolescent, measurement invariance

## Abstract

Emotion regulation (ER) is a basic psychological process that has been broadly linked to psychosocial adjustment. Due to its relationship with psychosocial adjustment, a significant number of instruments have been developed to assess emotion regulation in a reliable and valid manner. Among these, the *Emotion Regulation Questionnaire* (ERQ; Gross and John, 2003) is one of the most widely used, having shown good psychometric properties with adult samples from different cultures. Studies of validation in children and adolescents are, however, scarce and have only been developed for the Australian and Portuguese populations. The aim of this study was to validate the Spanish version of the ERQ for use in adolescents and determine possible differences according to the gender and age of young people. The sample consisted of 2060 adolescents (52.1% boys). Exploratory and Confirmatory factor analysis (EFA and CFA), multi-group analysis and Two-way multivariate analysis of variance (MANOVA) were performed and the percentiles calculated. The results of the AFE and CFA corroborated the existence of two factors related to the emotion regulation strategies of cognitive reappraisal and expressive suppression, showing acceptable internal consistency and test-retest reliability. Both factors also showed good criterion validity with personality traits, self-esteem, and social anxiety. Differences in cognitive reappraisal were found with regard to age, with younger students exhibiting the greatest mastery of this strategy. Gender differences were observed regarding the expressive suppression strategy, with boys being more likely to use this strategy than girls. A gender-age interaction effect was also observed, revealing that the use of the expressive suppression strategy did not vary by age in girls, and was more widely used by boys aged 12–14 years than those aged 15–16 years. However, we found evidence of measurement invariance across sex and age groups. The results suggest that the ERQ is a valid and reliable instrument that can be used to evaluate emotion regulation strategies in adolescents.

## Introduction

The scientific literature on emotion regulation (hereafter ER) has increased exponentially in recent years, giving rise to a large number of theories, methods, and techniques to examine the regulatory processes of emotion, which have proven to be broadly related to psychosocial adjustment (Gross, 2013). However, most studies have focused on adulthood due to the lack of instruments that assess ER specifically in childhood and adolescence. The aim of this study was to validate the Spanish version of the *Emotion Regulation Questionnaire* (ERQ; Cabello et al., 2012, for the original English version see Gross and John, 2003) for use in adolescents and to determine possible gender and age differences in this population.

Studies that have examined psychosocial adjustment in relation to ER have linked the development of these strategies to a higher quality of subjective well-being, less stress, and better social relationships (Fernández-Berrocal and Extremera, 2008; English et al., 2012; Teixeira et al., 2014; Beath et al., 2015). Having a good capacity for ER has also been shown to be a precursor of more effective cognitive processing (Ochsner and Gross, 2008); a capacity which in turn promotes the efficient use of ER strategies associated with good adjustment, such as cognitive reappraisal (Opitz et al., 2014). Moreover, there is broad consensus on the importance of managing emotions in an adaptive and socially appropriate way to promote adequate mental health (Southam-Gerow and Kendall, 2002; Westermann et al., 2013; Woodward et al., 2015).

One of the theoretical ER models with the most empirical support is the process model of emotion regulation proposed by Gross (Gross, 1998a,b, 2001, 2008, 2014; Gross and Thompson, 2007). This model defines ER as “the processes by which we influence which emotions we have, when we have them, and how we experience and express these emotions” (Gross, 1998b). Such processes can be automatic or controlled, conscious or nonconscious, but are always preceded by the activation of one's own or another's goal, hence they can also be categorized as extrinsic (interpersonal) or intrinsic (intrapersonal) (Gross and Thompson, 2007). Gross (2001) identified five types of ER strategies that can be classified based on whether they focus on antecedents or the emotional response itself. Antecedent-focused strategies regulate the emotional response by modifying incoming information before the emotion has been generated. This broader strategy includes four more specific strategies: situation selection, situation modification, attentional deployment, and cognitive change. In contrast, response-focused strategies, among them expressive suppression, involve influencing at least one of the three components of the emotional response (expressive, cognitive, or physiological) once the emotion has been elicited. However, empirical work based on Gross' theoretical model of emotion regulation has primarily focused on analyzing one of the forms of cognitive change, such as reappraisal and expressive suppression. This is due to the fact that these strategies are used more widely in everyday life, can be manipulated experimentally in the laboratory setting, and their study provides both antecedent-focused and response-focused strategies (Gross and John, 2003). Cognitive reappraisal is a strategy that involves cognitively construing information in such a way as to reinterpret the situation (by modulating its emotional meaning or analyzing our ability to cope with it) with a view to decreasing or increasing the emotional expression. By contrast, the expressive suppression strategy influences the “nature” of the emotional response by decreasing or inhibiting the ongoing emotion-expressive behavior.

To assess individual differences in the use of cognitive reappraisal and expressive suppression and their relationship with psychosocial adjustment, Gross and John (2003) designed the ERQ. The ERQ comprises 10 Likert-type items distributed on two scales to measure the degree of agreement regarding the use of both strategies. In their study, the authors reported the adequate internal consistency of both scales (α = 0.79 for cognitive reappraisal; α = 0.73 for expressive suppression) and their high test-retest reliability (*r* = 0.69). They also showed the convergent and discriminant validity of the instrument and found gender differences in the use of both types of ER. Following its publication, the questionnaire has been validated in numerous adult populations, providing ample proof of its adequate reliability and validity in all of them. Several studies have been conducted to validate the Spanish (Cabello et al., 2012), Italian (Balzarotti et al., 2010), German (Abler and Kessler, 2009), French (Christophe et al., 2009), and British (Ioannidis and Siegling, 2015) versions of the questionnaire. Other studies have compared the questionnaire's factorial structure in different ethnic and cultural groups, finding positive results when analyzing factorial invariance (Matsumoto et al., 2008; Melka et al., 2011; Sala et al., 2012).

Studies that have used the ERQ to examine these ER strategies with regard to adult psychosocial adjustment have shown that the frequent use of expressive suppression is negatively associated with emotional attention, clarity, and release, as well as mood repair efforts (John and Gross, 2004). Moreover, individuals who use the expressive suppression strategy to a greater extent exhibit less experiences and expressions of positive emotions, as well as lower levels of well-being, self-esteem, social support, and quality social relationships, as well as higher levels of negative emotionality, feelings of inauthenticity, and depressive symptoms. By contrast, the habitual use of cognitive reappraisal appears to be associated with more positive emotional experiences, closer interpersonal relationships, and greater willingness to seek social support and connect with peers, as well as enhanced well-being (Gross and John, 2003; Gross and Thompson, 2007; Nezlek and Kuppens, 2008; Balzarotti et al., 2010; Cabello et al., 2012; English et al., 2012). A positive relationship between trait anxiety and expressive suppression has also been found, where the relationship between this psychopathological trait and cognitive reappraisal is negative (Christophe et al., 2009).

Social anxiety is one of the most widely studied disorders in relation to the possible ER deficit associated with this condition (Jazaieri et al., 2015). Individuals diagnosed with this condition have reported greater use of expressive suppression, but less self-efficacy in implementing this strategy and difficulties in cognitively reappraising situations that have a large emotional impact (Werner et al., 2011; Aldao et al., 2014). This difficulty has been described as one of the causal factors of the disorder, as cognitive behavioral therapy has been shown to significantly mitigate the symptoms of social anxiety by reducing the use of expressive suppression and increasing the use of cognitive reappraisal in particular (Goldin et al., 2014).

The Big Five personality factors have also been widely discussed in relation to both ER strategies, with most studies reporting a positive relationship between all the traits, except neuroticism and cognitive reappraisal, and a negative relationship with expressive suppression. Neuroticism does not seem to bear a direct relationship with any of the ER strategies, as its relationship with these strategies is null or negative (Gross and John, 2003; Balzarotti et al., 2010; Cabello et al., 2012).

As for gender differences, most studies suggest that males tend to exhibit greater expressive suppression, although no differences have been found between males and females regarding the use of cognitive reappraisal (Gross and John, 2003; Christophe et al., 2009; Balzarotti et al., 2010; Melka et al., 2011).

Most of these results refer to higher education or adult populations, with few studies having explored the relationship between these two ER strategies and psychosocial adjustment at younger ages. Although adolescence is the stage where emotions and regulating processes play a fundamental role (Gross and Thompson, 2007), the process has not been sufficiently explored in this age group due to the lack of tools that have been culturally adapted to assess ER in this period. In this line, Gullone and Taffe (2012) in Australia, and Teixeira et al. (2014) in Portugal validated the ERQ for its use in children and adolescents. Both studies demonstrated the good psychometric properties of the questionnaire whose factorial structure was similar to that shown in adults. In this regard, results in young children have shown that both strategies are related to psychological adjustment and personality, and linked the greater use of the expressive suppression strategy with more depressive symptoms, higher neuroticism, and less self-esteem, well-being, extraversion, agreeableness, and conscientiousness. Conversely, the increased use of the cognitive reappraisal strategy has been associated with lower neuroticism and less depressive symptoms, and greater extraversion, agreeableness, conscientiousness, and openness to experience, as well as higher levels of self-esteem and well-being. Both studies revealed gender differences in the use of ER strategies, with boys engaging more in expressive suppression. The study of Teixeira et al. (2014), however, is the only one to find differences according to age, with younger students reporting a greater use of both strategies. In spite of this, these authors found evidences of measurement invariance across gender and age (Teixeira et al., 2014).

## The present study

Despite the above, previous studies have only validated the ERQ for use in children with Australian and Portuguese samples, and it remains unknown whether this instrument is suitable for use in adolescents in other countries such as Spain, where the language is different and the instrument has proved to be suitable for its use in adults.

The aim of this study was to explore ER in Spanish adolescents through the validation of the ERQ instrument. This general objective can be broken down into four more specific objectives:

1. Determine the psychometric properties (test-retest reliability, internal consistency, and criterion validity) and establish the factorial structure of the Spanish version of the ERQ in Spanish adolescents. Criterion validity was assessed by analyzing the strength of the relationship between the factors of the ERQ and measures of social anxiety, positive self-esteem, and personality traits. The relationship between these measures and the ER construct has been amply demonstrated (John and Gross, 2004; Cabello et al., 2012; Gullone and Taffe, 2012; Aldao et al., 2014; Teixeira et al., 2014; Jazaieri et al., 2015).
2. To test measurement invariance of ERQ in order to evaluate if the measured constructs have the same meaning across subgroup samples (gender and age), regardless of group membership.
3. Analyze possible differences in ER among adolescents according to gender and age or the interaction of both variables.
4. Describe the level of ER in youth through the analysis of percentiles.

The following hypotheses have been formulated in relation to the above objectives.

- H1: The ERQ will have the same factorial structure as demonstrated in the validation of the questionnaire with adults and exhibit good test-retest reliability and internal consistency.
- H2: The ERQ instrument will also demonstrate adequate criterion validity (Cabello et al., 2012). On the one hand, we expect to find a direct relationship between expressive suppression and cognitive and behavioral manifestations of social anxiety (Werner et al., 2011; Aldao et al., 2014), negative self-esteem (Teixeira et al., 2014), and neuroticism (Gullone and Taffe, 2012). Moreover, we expect to find a positive relationship between cognitive reappraisal and all personality traits, except neuroticism (Gross and John, 2003; Balzarotti et al., 2010; Cabello et al., 2012; Gullone and Taffe, 2012) and positive self-esteem (Teixeira et al., 2014). On the other hand, we think there will be an inverse relationship between cognitive reappraisal and the dimensions of social anxiety analyzed, neuroticism, and negative self-esteem, as well as between the expressive suppression strategy and the other personality traits and positive self-esteem (Gross and John, 2003; Balzarotti et al., 2010; Werner et al., 2011; Cabello et al., 2012; Gullone and Taffe, 2012; Aldao et al., 2014; Teixeira et al., 2014).
- H3: We will find evidence of measurement invariance across gender and age (Teixeira et al., 2014).
- H4: We expect to observe significant differences in expressive suppression by gender, with girls making the least use of the expressive suppression strategy (Gross and John, 2003; Christophe et al., 2009; Melka et al., 2011; Gullone and Taffe, 2012).
- H5: With respect to age, it is expected that younger adolescents will use both ER strategies more frequently (Teixeira et al., 2014).

## Materials and methods

### Participants

The reference population used to conduct the study was all students enrolled in compulsory secondary education (ESO, *Educación Secundaria Obligatoria* in Spanish) in Andalusia (an autonomous region in the south of Spain). To select the sample, stratified, single-stage, random cluster sampling with proportional allocation was performed (Cea D'Ancona, 2004). The strata were geographic area, school ownership (public or private), and type of municipality in which the school was located.

A confidence level of 95.5% and a sample error of 2.5% was applied. High variability was assumed (*p* = *q* = 0.5) (Cea D'Ancona, 1996).

The final sample comprised 2060 ESO students. Of these, 52.1% were boys and 47.7% were girls. The age of the students ranged from 12 to 19 years old (*M* = 14.34; *SD* = 1.34). As regards school grade, 28.4% of the students were in the first year of ESO, 28.4% were in the second year, 22.1% in the third year, and 21.1% in the fourth year.

### Instruments

The ERQ (Gross and John, 2003; Spanish version validated by Cabello et al., 2012) is a self-report consisting of 10 Likert-type items measured according to the degree of agreement with the items (1 = *completely disagree*; 7 = *completely agree*). Validations of the ERQ in Spanish adults and non-Spanish teenagers or children have shown good psychometric properties, as they replicated the original factorial structure through two factors related to two ER strategies: cognitive reappraisal and expressive suppression (Cabello et al., 2012; Gullone and Taffe, 2012; Teixeira et al., 2014).

The *Rosenberg Self-Esteem Scale* (RSES; Rosenberg, 1965) consists of 10 items measured on a 1-4 point Likert-type scale measured according to degree of agreement. Recent studies that have analyzed the factorial structure of the RSES in Spanish adolescents have shown the existence of two factors referring to negative self-esteem and positive self-esteem (Gómez-Ortiz et al., in press). Our study has shown the RSES to have acceptable internal consistency: Ωpositive self-esteem = 0.83 and Ωnegative self-esteem = 0.83.

The *Social Anxiety Scale for Adolescents* (SAS-A; La Greca and López, 1998) was validated in Spanish adolescents by Olivares et al. (2005). The scale consists of 18 items measured on a 5-point Likert-type scale rated according to the frequency with which the subject has experienced the symptoms described in the questionnaire (1 = *not at all*, 5 = *all the time*). The scale assesses three factors of social anxiety. The first, called “fear of negative evaluation,” measures fears, concerns, or worries regarding peers' negative evaluations and includes eight items; the second, called “social avoidance and distress in new situations” measures social fears and the difficulty associated with new social situations or interactions with strangers and consists of six items; while the last factor, “generalized social avoidance and distress”, measures discomfort and more general social inhibition, and comprises four items. In our study, the questionnaire has shown acceptable internal consistency as evaluated by McDonald's omega (0.92 for the general scale, 0.89 for fear of negative evaluation, 0.87 for fear of new situations, and 0.84 for fear of general situations).

The *Single-item measures of personality* (SIMPs; Woods and Hampson, 2005) questionnaire assesses personality using five bipolar statements describing the Big Five personality traits (extraversion, agreeableness, conscientiousness, neuroticism, and openness/intellect) and their opposite traits. The questionnaire uses a response scale comprising a nine-point graded line placed between two descriptions, thus allowing respondents to choose which of the two poles best describes them. Hence, a middle score indicates that both descriptions describe the respondent equally well, while a score closer to pole A or B indicates that this description best describes the respondent.

### Procedure

After obtaining permission from the administrative bodies of the schools, the battery of questionnaires were administered to a representative sample of the population of young students attending ESO in Andalusia. At all times, the students were informed of the anonymous, confidential, and voluntary nature of their participation and any doubts that arose were clarified. After 1 month, which is the optimal time recommended for the analysis of test-retest reliability (Cea D'Ancona, 1996), the ERQ questionnaire was re-administered to a subsample comprising 443 students from five different schools. The study was carried out in accordance with the Declaration of Helsinki. Ethics approval was obtained from the Research Ethics Committee of the University of Cordoba, Spain.

### Statistical analysis

At first, descriptive analyses of each item of the ERQ were performed. Following the recommendations made by Neukrug and Fawcett (2014) to validate questionnaires, the sample was divided into two parts, taking gender as the selection variable with a proportional number of boys and girls. This was done in order to proceed with the exploratory (EFA) and confirmatory (CFA) factor analyses by means of cross-validation, optimizing the generalization of the model by using different subsamples (Delgado-Rico et al., 2012). To obtain evidence concerning the dimensionality of the *ERQ*, an EFA was performed using the Factor 9.3. statistical software (Lorenzo-Seva and Ferrando, 2006), adopting the unweighted least-squares (ULS) estimation method, the Promin rotation method, and based on the polychoric correlation matrix, recommended when working with non-normal distribution samples and when the measurement instrument involves ordinal items (Bryant and Satorra, 2012). To find out the validity based on the instrument's internal structure and to confirm the factorial structure generated by the EFA, a confirmatory factor analysis (CFA) using the Maximum Likelihood estimation method with robust correction was performed. This approach is recommended for samples with a non-normal distribution and when the univariate distributions of ordinal items are asymmetric or show excessive kurtosis (Bryant and Satorra, 2012). The model fit was assessed considering the chi-square significance value of Satorra-Bentler (X^2^S-B) (values greater than 0.01 indicate a good fit). However, because the value of this index is subject to other variables such as sample size, other indicators were considered. These included the comparative fit index (CFI), the non-normed fit index (NNFI) (values greater than 0.90 indicate a good fit), the standardized root mean square residual (SRMR), and the root mean square error of approximation (RMSEA) (values less than 0.08 indicate a good fit) (Hu and Bentler, 1999; Byrne, 2014).

Multi-group factor analysis was performed to evaluate the generalization of the model across the different sex and age samples. This kind of testing includes a number of sequential analyses with progressively restricted models. We presented four different models to perform model comparison tests: model 1, where the same factor structure is imposed to groups (configural invariance); model 2, where covariances are constrained to be equal across groups; model 3 where factor loadings are constrained to be equal across groups (metric invariance); and model 4 where factor loadings and covariances are constrained to be equal across groups (residual invariance) (Byrne et al., 1989). The chi square difference test (Δχ^2^) is often used to test the invariance degree (Bollen, 1989). Non-significant changes in chi square suggest inter-groups invariance. However, instead of relying on just one index it is recommended the use of other fit measures (NNFI, CFI, RMSEA, and SRMR). The cutoff point suggested in the literature to accept the hypothesis of invariance across groups is a change of ≤ 0.01 (Dimitrov, 2010). Multi-group and CFA were performed using EQS 6.2.

Given the characteristics of the variables and the absence of multivariate normality, the analysis of internal consistency was based on the results of McDonald's omega (Elosua Oliden and Zumbo, 2008), which was calculated using Factor 9.3 software.

To examine criterion validity and the temporal stability of the instrument, Pearson's correlation coefficient was used. To determine the relationship between the dimensions of emotional self-regulation and gender and age, a Two-way (gender) multivariate analysis of variance (MANOVA) was also performed (the age of the participants was divided into two ranges to distinguish two periods of adolescence: early adolescence from 12 to 14 years of age, and middle adolescence from 15–16 years of age; Smetana et al., 2006). Finally, the percentiles were calculated for each of the factors of the questionnaire. The latter analyses were performed with SPSS 18.0 software.

## Results

Media, standard deviation, skewness, kurtosis for each item are showed in Table 1. The highest media was 5.24 (“*When I'm faced with a stressful situation, I make myself think about it in a way that helps me stay calm*”) and the lowest 3.3 (“*When I am feeling positive emotions, I am careful not to express them*”).

**Table 1:** **Descriptive univariate analysis and factor loadings and communalities of EFA**.

**Item**	**M**	***SD***	**Skewness**	**Kurtosis**	**F1**	**F2**	**Com**.
1. I keep my emotions to myself / Guardo mis emociones para mí mismo	4.23	2.03	−0.19	−1.18	0.58		0.33
2. When I am feeling positive emotions, I am careful not to express them / Cuando estoy sintiendo emociones positivas, tengo cuidado de no expresarlas	3.3	2.01	0.36	−1.12	0.70		0.46
3. I control my emotions by not expressing them / Controlo mis emociones no expresándolas	3.79	1.95	0.04	−1.09	0.88		0.73
4. When I am feeling negative emotions, I make sure not to express them / Cuando estoy sintiendo emociones negativas, me aseguro de no expresarlas	4.56	1.96	−0.32	−1.02	0.41		0.34
5. When I want to feel more positive emotion (such as joy or amusement), I change what I'm thinking about./ Cuando quiero incrementar mis emociones positivas (p.ej. alegría, diversión), cambio el tema sobre el que estoy pensando	5.05	1.71	−0.81	0.01		0.58	0.27
6. When I want to feel less negative emotion (such as sadness or anger), I change what I'm thinking about / Cuando quiero reducir mis emociones negativas (p.ej. tristeza, enfado), cambio el tema sobre el que estoy pensando.	4.97	1.9	−0.75	−0.47		0.60	0.31
7. When I'm faced with a stressful situation, I make myself think about it in a way that helps me stay calm /Cuando me enfrento a una situación estresante, intento pensar en ella de un modo que me ayude a mantener la calma.	5.24	1.68	−0.91	0.15		0.49	0.22
8. When I want to feel more positive emotion, I change the way I'm thinking about the situation / Cuando quiero incrementar mis emociones positivas, cambio mi manera de pensar sobre la situación.	4.59	1.75	−0.47	−0.46		0.68	0.49
9. I control my emotions by changing the way I think about the situation I'm in / Controlo mis emociones cambiando mi forma de pensar sobre la situación en la que me encuentro	4.55	1.7	−0.42	−0.43		0.65	0.49
10. When I want to feel less negative emotion, I change the way I'm thinking about the situation/ Cuando quiero reducir mis emociones negativas, cambio mi manera de pensar sobre la situación	4.83	1.75	−0.57	−0.39		0.68	0.46

*Only factor loadings >0.30 are shown: Com, communalities*.

In what follows, we present the results of the ERQ validation.

The Kaiser-Meyer-Olkin (KMO) measure of sampling adequacy, with a value of 0.78, and the statistically significant Bartlett's test of sphericity, *X*^2(45)^ = 2024.7; *p* < 0.01, confirmed the benefits of conducting an EFA. Two factors were applied in this analysis, one for each of the dimensions measured, where the total explained variance was 52.36%. Modifying the number of factors returned no conclusive results. Table 1 shows the factor loadings and communalities for each item.

The first factor, “expressive suppression,” yielded an explained variance of 35.61% and comprised four items related to the skill to decrease or inhibit the emotion-expressive behavior. The second factor, “cognitive reappraisal,” with an explained variance of 11.26%, was made up of six items that describe the ability to regulate emotions by cognitively modifying the situation linked to creating the feeling.

**Figure 1:**
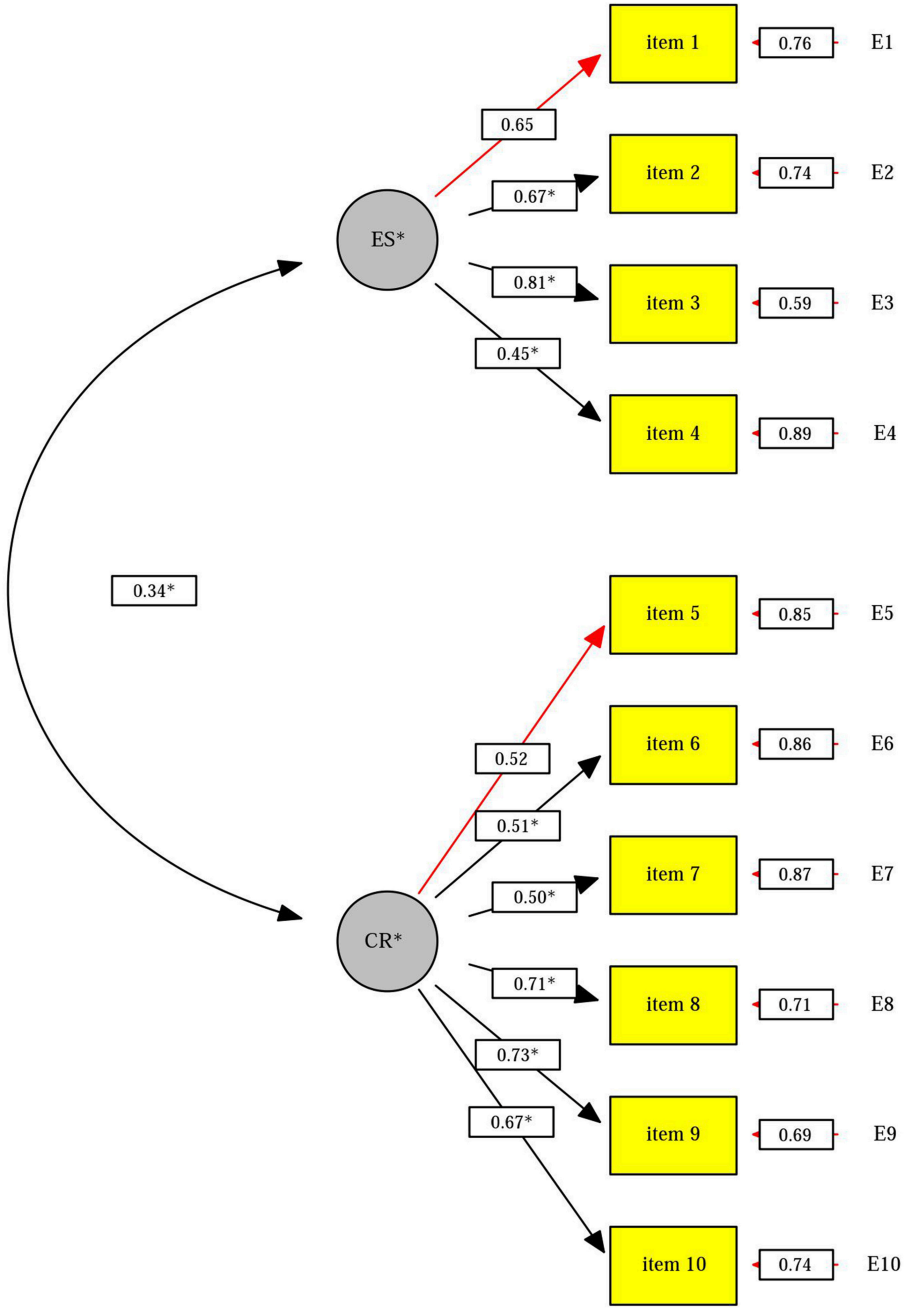
**Normalized CFA coefficients in the ERQ items**. Note: CR, cognitive reappraisal; ES, expressive suppression. ^*^p < 0.05.

The results of the CFA verified the original factorial structure of two factors (cognitive reappraisal and expressive suppression), showing the following fit indices: X^2^ S-B = 221.46 (34); *p* = 0.00; NNFI = 0.91; CFI = 0.93; SRMR = 0.07; RMSEA = 0.07. Moreover, the factor loadings and the correlation between the factors were statistically significant. The parameters of the proposed model are shown in Figure 1.

In the multi-group analysis four more progressively restricted models were compared. Chi-square differences were non-significant between models 1 and 2, between models 1 and 3 and between models 1 and 4. Changes on CFI, NNFI, RMSEA, and SRMR were minimal between models 1 and 2 and between models 1 and 3, and 1 and 4 (see Tables 2, 3). These results seem to present reasonable evidence of measurement invariance across sex and age groups.

**Table 2:** **ERQ Multi-group analysis in boys and girls: metric and residual invariance**.

**Models**	**χ^2^S-B**	***df***	***P***	**NNFI**	**CFI**	**RMSEA**	**SRMR**	**Δχ^2^S-B**	***p***	**Δ*df***	**ΔNNFI**	**ΔCFI**	**ΔRMSEA**	**ΔSRMR**
Model 1	437.54	68	0.00	0.91	0.93	0.07	0.07							
Model 2	441.68	69	0.00	0.91	0.93	0.07	0.07	4.14	1	1	0.00	0.00	0.00	0.00
Model 3	444.61	76	0.00	0.92	0.93	0.07	0.07	7.07	1	8	0.01	0.00	0.00	0.00
Model 4	448.70	77	0.00	0.92	0.93	0.07	0.07	11.16	1	9	0.01	0.00	0.00	0.00

*Model 1, without constraints; Model 2, constrained covariances; Model 3, constrained factor loadings; Model 4, constrained factor loadings and covariances*.

**Table 3:** **ERQ Multi-group analysis in students under 15 years and students aged 15 and older: metric and residual invariance**.

**Models**	**χ^2^S-B**	***df***	***p***	**NNFI**	**CFI**	**RMSEA**	**SRMR**	**Δχ^2^S-B**	***p***	**Δ*df***	**ΔNNFI**	**ΔCFI**	**ΔRMSEA**	**ΔSRMR**
Model 1	435.61	68	0.00	0.91	0.93	0.07	0.07							
Model 2	436.86	69	0.00	0.91	0.93	0.07	0.07	1.25	1	1	0.00	0.00	0.00	0.00
Model 3	447.54	76	0.00	0.92	0.93	0.07	0.07	11.93	1	8	0.01	0.00	0.00	0.00
Model 4	448.52	77	0.00	0.92	0.93	0.07	0.07	12.91	1	9	0.01	0.00	0.00	0.00

*Model 1, without constraints; Model 2, constrained covariances; Model 3, constrained factor loadings; Model 4, constrained factor loadings and covariances*.

To assess the reliability of the instrument, internal consistency and test-retest reliability were analyzed. With regard to internal consistency, a McDonald's omega of 0.75 and 0.78 was obtained for the expressive suppression and cognitive reappraisal scales, respectively. In the total scale, the McDonald's omega was 0.75. The test-retest correlations showed significant and positive values for each of the instrument scales (see Table 4).

**Table 4:** **Test-retest reliability and criterion validity of the ERQ scales**.

	**Expressive suppression**	**Cognitive reappraisal**
Test-retest reliability	0.55^***^	0.44^***^
**SELF-ESTEEM**		
Positive self-esteem	−0.098	0.218^***^
Negative self-esteem	0.178^***^	−0.047^*^
**SOCIAL ANXIETY**		
Fear of negative evaluation	0.156^***^	0.081^***^
Fear of new situations	0.243^***^	0.066^**^
General social anxiety	0.307^***^	−0.003
**PERSONALITY TRAITS**		
Extraversion	−0.150^**^	−0.036
Agreeableness	0.099	0.082
Neuroticism	−0.109	−0.005
Conscientiousness	−0.024	0.09
Openness to experience	0.023	0.175^**^

* p < 0.05;

** p < 0.01;

*** *p = 0.000*.

As regards the analysis of criterion validity, correlation analysis showed a statistically significant and direct relationship between cognitive reappraisal and positive self-esteem, as well as negative self-esteem and expressive suppression. With regard to social anxiety, a positive and significant relationship between all factors of this construct and expressive suppression was observed. For cognitive reappraisal, a positive and significant relationship was only found with fear of negative evaluation and new situations. As for the correlation with personality traits, the only remarkable finding was the inverse relationship between extraversion and expressive suppression and the positive relationship between openness to experience and cognitive reappraisal (see Table 4).

**Figure 2:**
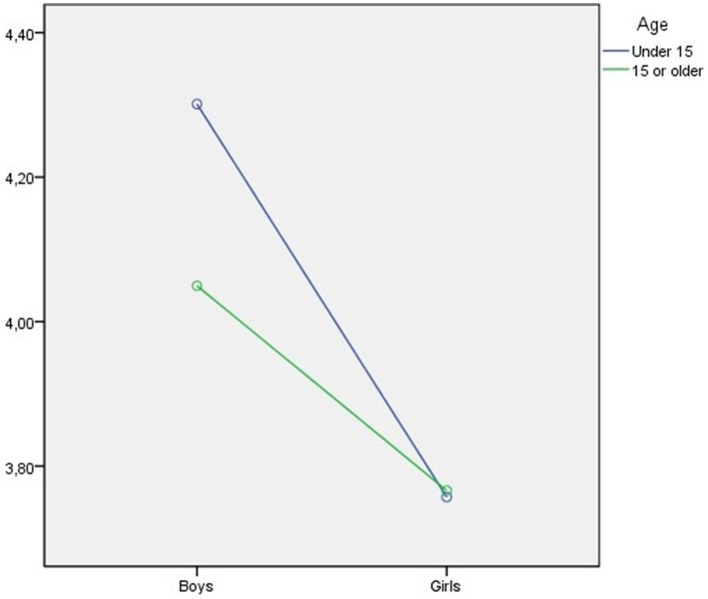
**Effect of interaction of gender and age on expressive suppression strategy**.

The MANOVA, which was performed to examine gender and age differences, indicated the important effect of gender only for the expressive suppression dimension *F*_(1, 1954)_ = 37.67, *p* < 0.01; and significant differences as a function of age only for the cognitive reappraisal dimension *F*_(1, 1928)_ = 17.7, *p* < 0.01. The interaction of both variables was not found to have a significant effect on the cognitive reappraisal dimension, although a significant effect was found for the expressive suppression dimension *F*_(1, 1954)_ = 3.93, *p* = 0.048. The univariate analyses indicated significant differences in expression suppression between girls and boys, with boys using this strategy more than girls (Cohen's D = 0.27). Significant differences in cognitive reappraisal in terms of age were also found, with younger students stating that they used this strategy more frequently (Cohen's D = 0.19). As regards the effect of the interaction, the analysis indicated that girls obtain practically the same score in the expressive suppression dimension, regardless of their age. Nonetheless, the girls' scores were lower than that of the boys, who use this strategy to a larger extent, especially those in the 12–14 age range (see Table 5). Figure 2 shows the effect of the gender-age interaction in the expressive suppression dimension.

**Table 5:** **Means and standard deviations of the two emotion regulation strategies for the total sample and by gender and age**.

		**Expressive suppression**	**Cognitive reappraisal**
Total sample		3.97 (1.44)	4.86 (1.13)
Boys		4.15 (1.42)	4.89 (1.15)
Girls		3.76 (1.44)	4.83 (1.21)
Under 15 (−15)		4.02 (1.48)	4.97 (1.16)
15 years and older (15+)		3.91 (1.40)	4.76 (1.11)
Boys	−15	4.29 (1.46)	5.02 (1.18)
	15+	4.03 (1.37)	4.78 (1.12)
Girls	−15	3.76 (1.45)	4.93 (1.13)
	15+	3.76 (1.43)	4.73 (1.10)

Finally, we calculated the percentiles for each of the ER strategies taking into account the gender and age differences obtained in the MANOVA (see Table 6).

**Table 6:** **Percentiles of each of the ER strategies**.

	**Expressive suppression (Boys)**	**Expressive suppression (Girls)**	**Cognitive reappraisal (under 15)**	**Cognitive reappraisal (15 years and older)**
10	2.25	1.75	3.5	3.46
20	3	2.5	4	4
25	3.25	2.75	4.33	4.16
30	3.5	3	4.5	4.23
40	4	3.25	4.66	4.5
50	4.25	3.75	5	4.83
60	4.5	4	5.33	5
70	5	4.5	5.66	5.33
75	5.25	4.75	5.83	5.5
80	5.50	5	6	5.66
90	6	5.75	6.5	6.16

## Discussion

We used a sample of 2060 students enrolled in compulsory secondary education (ESO) to validate the Spanish version of the ERQ in adolescents. The convergent and discriminant validity of the instrument, as well as possible differences according to the gender and age of the adolescents were also analyzed. The results highlighted the good psychometric properties of the questionnaire, as well as the presence of gender differences in the expressive suppression factor and age differences in the cognitive reappraisal dimension. Moreover, an interaction effect of both variables was observed in the first factor.

The first objective of the study was to determine the psychometric properties of the ERQ in Spanish adolescents. As we stated in our first hypothesis, the factorial structure analysis confirmed the existence of two independent factors related to cognitive reappraisal and expressive suppression strategies. This finding is consistent with the results of previous studies that have validated the questionnaire for use in adults (Gross and John, 2003; John and Gross, 2004; Christophe et al., 2009; Balzarotti et al., 2010; Melka et al., 2011; Cabello et al., 2012; Sala et al., 2012) and adolescents (Gullone and Taffe, 2012; Teixeira et al., 2014). As it was hypothesized, this patterns were remarkably invariant across different sex and age groups, in line with previous studies (Teixeira et al., 2014). Both factors showed adequate internal consistency and test-retest reliability.

With respect to the criterion validity of the questionnaire in relation to personality traits, the results partially support the second hypothesis, as not all personality traits showed a significant relationship with the ER strategies evaluated. Specifically, a positive relationship was found between cognitive reappraisal and openness to experience, whereas a negative relationship was observed between extraversion and expressive suppression. Contrary to what has been reported in some previous studies (Gross and John, 2003; Balzarotti et al., 2010), we found no relationship between the other personality traits and each of the strategies. However, our study is in line with others that have not found a significant relationship between each of the personality traits and both ER strategies (Cabello et al., 2012; Gullone and Taffe, 2012; Ioannidis and Siegling, 2015). Nonetheless, the analysis of the relationship between the dimensions of the ERQ and self-esteem was entirely consistent with our second hypothesis linking positive self-esteem with increased use of cognitive reappraisal and less frequent use of expressive suppression. An inverse relationship was observed between negative self-esteem and the use of both strategies, that is, greater use of expressive suppression and less use of cognitive reappraisal (John and Gross, 2004; Teixeira et al., 2014).

Moreover, our results confirm the second hypothesis, as we found a moderate relationship between the use of expressive suppression and general social anxiety and other cognitive and behavioral manifestations of this problem, such as fear of negative evaluation and new situations (Werner et al., 2011; Ioannidis and Siegling, 2015). Contrary to this hypothesis, however, the use of the cognitive reappraisal strategy showed a weak but significant relationship with fear of negative evaluation and new situations. These results could indicate that although adolescents who suffer from this problem or related social fears use cognitive reappraisal, they do not appear to have the ability to properly use this strategy and therefore do not benefit from its positive effects (Jazaieri et al., 2015). In this regard, it has been shown that encouraging cognitive reappraisal, which improves the self-efficacy of this ability in patients, contributes to mitigating symptoms of social anxiety, even 1 year after the intervention (Goldin et al., 2012). For this reason, several studies recommend applying cognitive behavioral therapy as it has been proven to minimize the symptoms and emotional impact deriving from social anxiety, and teach those affected to effectively use cognitive reappraisal and control the use of expressive suppression (Aldao et al., 2014; Goldin et al., 2014).

The above results indicate that, in general, the frequent use of expressive suppression appears to be linked to worse adjustment outcomes. Although negative indicators have been associated with the use of cognitive reappraisal, this strategy is most often associated with qualities or behaviors related to positive psychosocial adjustment (Gross and John, 2003; Gullone and Taffe, 2012; Teixeira et al., 2014). Given that the cognitive reappraisal strategy allows anticipating the emotional consequences of a particular situation, individuals who use this strategy may not need to experience the emotion itself to regulate their emotional response, rather, the anticipation itself allows them to act accordingly depending on the moment and the particular social demands of the situation. In contrast, individuals who use the expressive suppression strategy exert cognitive and behavioral control that is expressed only at the behavioral level, thus maintaining the subjective emotional experience. The psychosocial effects of the manifestation of that emotion could therefore be especially harmful when it comes to negative emotions like sadness, fear, or anger (John and Gross, 2004; Cabello et al., 2012). In this regard, our study highlights the relationship between increased use of cognitive reappraisal and positive self-esteem and openness to experience, although it has also been linked with certain social fears that are very present in this life stage where social acceptance is a priority (LaFontana and Cillessen, 2009). Moreover, the frequent use of expressive suppression is related to and appears to favor negative self-esteem and introversion, as well as the development of behaviors and thoughts associated with social anxiety.

In terms of the second objective, the results indicated gender differences in expressive suppression, as stated in the third hypothesis. Specifically, boys were found to use the expressive suppression strategy more frequently, although no gender differences were found regarding the use of the cognitive reappraisal strategy, in line with previous studies (Gross and John, 2003; Christophe et al., 2009; Balzarotti et al., 2010; Melka et al., 2011; Cabello et al., 2012; Gullone and Taffe, 2012; Teixeira et al., 2014). Aside from the gender differences widely established in the neurosciences for the psychology of emotions, this result may be explained by the process of socialization and cultural norms transmitted from childhood that seek to establish social gender roles, which lead boys to suppress or inhibit their emotional expression (Brody and Hall, 2000).

As regards age differences, the results support the fourth hypothesis, as we found that younger adolescents (those under the age of 15) exhibited a greater mastery of the cognitive reappraisal strategy than older adolescents (those 15 and older). Gender and age were also observed to have an interaction effect on the expressive suppression dimension. However, age differences regarding the use of expressive suppression were only found in boys, with those younger than 15 using this strategy more frequently. Although the transversal nature of our study precludes drawing conclusions about the development of the ER process, these results are partially consistent with the previous literature (Gullone et al., 2010; Teixeira et al., 2014). Indeed, they may indicate that older students, who are fully immersed in this complex stage of life and more aware of the challenges facing them, experience situations more intensely and are subject to greater emotional variability and therefore find it more difficult to use the appropriate ER strategies unlike younger adolescents, who have not yet reached this stage and deal with all types of situations in a more emotionally stable manner, which helps them respond in a more emotionally regulated manner (Smetana et al., 2006).

Finally, with regard to the third objective, the analysis of percentiles taking into account the existing gender and age differences, indicated that 50% of young people obtain a score ranging from 3.25 to 5.25 (boys) and from 2.75 to 4.75 (girls) in the expressive suppression strategy. The cognitive reappraisal scores range from 4.33 to 5.83 in the case of those under 15, and from 4.16 to 5.5 for adolescents aged 15 years or older. Since we have found no previous studies that describe the distribution of scores of young people in each dimension, it is not possible to establish a comparative framework.

### Limitations and future directions

This study is not without its limitations. Although we provide evidence of significant gender and age differences, and relationships between the two ER strategies and measures of positive and negative adjustment, its transversal nature makes it impossible to establish causal relationships. In this sense, it would be necessary to conduct a longitudinal study to determine whether the relationship between these variables and individual differences remain unchanged over time.

It would also be of interest to determine which individual factors depend on developmental contexts that influence the use of both strategies and therefore condition adequate future psychosocial adjustment. This would allow us to work with and include such factors as the focus of intervention to improve ER skills in adolescents and ensure their welfare and future psychological health.

Despite these limitations, the results of this research have demonstrated in a large and representative sample of adolescents that the ERQ is a reliable and valid instrument for assessing ER in this population, which will be of great use in applied developmental research.

## Author contributions

All authors contributed to the interpretation of data, helped to draft and have revised the manuscript to get the final text.

### Conflict of interest statement

The authors declare that the research was conducted in the absence of any commercial or financial relationships that could be construed as a potential conflict of interest.

## References

[B1] Abler B. Kessler H. (2009). Emotion regulation questionnaire– eine deutschsprachige Fassung des ERQ von Gross und John [Emotion Regulation Questionnaire – A German-language version of the ERQ by Gross and John]. Diagnostica 55, 144–152. 10.1026/0012-1924.55.3.144

[B2] Aldao A. Jazaieri H. Goldin P. R. Gross J. J. (2014). Adaptive and maladaptive emotion regulation strategies: Interactive effects during CBT for social anxiety disorder. J. Anxiety Disord. 28, 382–389. 10.1016/j.janxdis.2014.03.005 24742755 PMC4089517

[B3] Balzarotti S. John O. P. Gross J. J. (2010). An italian adaptation of the emotion regulation questionnaire. Eur. J. Psychol. Assess. 26, 61–67. 10.1027/1015-5759/a000009

[B4] Beath A. P. Jones M. P. Fitness J. (2015). Predicting distress via emotion regulation and coping: measurement variance in trait EI scales. Pers. Individ. Dif. 84, 45–51. 10.1016/j.paid.2014.12.015

[B5] Bollen K. A. (1989). Structural Equations with Latent Variables. New York, NY: Wiley.

[B6] Brody L. R. Hall J. A. (2000). Gender, emotion, and expression, in Handbook of Emotions, eds Lewis M. Haviland J. M. (New York, NY: Guilford Press), 338–349.

[B7] Bryant F. B. Satorra A. (2012). Principles and practice of scaled difference Chi-Square testing. Struct. Eqn Model. 19, 372–398. 10.1080/10705511.2012.687671

[B8] Byrne B. (2014). Structural Equation Modeling With Lisrel, Prelis, and Simplis: Basic Concepts, Applications, and Programming (Multivariate Applications Series) (Reprint Edition). New Jersey, NJ: Psychology Press.

[B9] Byrne B. Shavelson R. J. Muthén B. (1989). Testing for the equivalence of factor covariance and means structures: the issue of partial measurement invariance. Psychol. Bull. 105, 456–466. 10.1037/0033-2909.105.3.456

[B10] Cabello R. Salguero J. M. Fernández-Berrocal P. Gross J. J. (2012). A spanish adaptation of the emotion regulation questionnaire. Eur. J. Psychol. Assess. 29, 234–240. 10.1027/1015-5759/a000150

[B11] Cea D'Ancona M. A. (1996). La selección de las unidades de observación: El diseño de la muestra, in Metodología cuantitativa: Estrategias y técnicas de investigación social ed Cea D'Ancona M. A. (Madrid: Síntesis), 159–215.

[B12] Cea D'Ancona M. A. (2004). Análisis multivariable. Teoría y Práctica en la Investigación Social. Madrid: Síntesis.

[B13] Christophe V. Antoine P. Leroy T. Delelis G. (2009). Assessment of two emotional regulation processes: expressive suppression and cognitive reevaluation. Rev. Eur. Psychol. Appl. 59, 59–67. 10.1016/j.erap.2008.07.001

[B14] Delgado-Rico E. Carretero-Dios H. Ruch W. (2012). Content validity evidences in test development: an applied perspective. Int. J. Clin. Health Psychol. 12, 449–459.

[B15] Dimitrov D. M. (2010). Testing for the factorial invariance in the context of construct validation. Meas. Eval. Couns. Dev. 43, 121–149. 10.1177/0748175610373459

[B16] Elosua Oliden P. Zumbo B. D. (2008). Coeficientes de fiabilidad para escalas de respuesta categórica ordenada. Psicothema 20, 896–901. 18940100

[B17] English T. John O. P. Srivastava S. Gross J. J. (2012). Emotion regulation and peer-rated social functioning: A 4-year longitudinal study. J. Res. Pers. 46, 780–784. 10.1016/j.jrp.2012.09.006 23471162 PMC3587109

[B18] Fernández-Berrocal P. Extremera N. (2008). A review of trait meta-mood research. Int. J. Psychol. Res. 2, 39–67.

[B19] Goldin P. R. Lee I. Ziv M. Jazaieri H. Heimberg R. G. Gross J. J. (2014). Trajectories of change in emotion regulation and social anxiety during cognitive-behavioral therapy for social anxiety disorder. Behav. Res. Ther. 56, 7–15. 10.1016/j.brat.2014.02.005 24632110 PMC4136443

[B20] Goldin P. R. Ziv M. Jazaieri H. Werner K. H. Kraemer H. Heimberg R. G. (2012). Cognitive reappraisal self-efficacy mediates the effects of individual cognitive-behavioral therapy for social anxiety disorder. J. Consult. Clin. Psychol. 80, 1034–1040. 10.1037/a0028555 22582765 PMC3424305

[B21] Gómez-Ortiz O. Casas C. Ortega-Ruiz R. (in press). Ansiedad social en la adolescencia: Factores psico-evolutivos y de contexto familiar. Behav. Psychol. Psicología Conductual.

[B22] Gross J. J. (1998a). Antecedent- and response-focused emotion regulation: divergent consequences for experience, expression, and physiology. J. Pers. Soc. Psychol. 74, 224–237. 10.1037/0022-3514.74.1.224 9457784

[B23] Gross J. J. (1998b). The emerging field of emotion regulation: an integrative review. Rev. Gen. Psychol. 2, 271–299. 10.1037/1089-2680.2.3.271

[B24] Gross J. J. (2001). Emotion regulation in adulthood: timing is everything. Curr. Dir. Psychol. Sci. 10, 214–219. 10.1111/1467-8721.00152

[B25] Gross J. J. (2008). Emotion Regulation, in Handbook of Emotions, eds Lewis M. Haviland-Jones J. M. Feldman Barrett L. (New York, NY: Guilford Press), 497–512.

[B26] Gross J. J. (2013). Emotion regulation: taking stock and moving forward. Emotion 13, 359–365. 10.1037/a0032135 23527510

[B27] Gross J. J. (2014). Emotion regulation: conceptual and empirical foundations, in Handbook of Emotion Regulation, ed Gross J. J. (New York, NY; London: Guilford Press), 3–20.

[B28] Gross J. J. John O. P. (2003). Individual differences in two emotion regulation processes: implications for affect, relationships, and well-being. J. Pers. Soc. Psychol. 85, 348–362. 10.1037/0022-3514.85.2.348 12916575

[B29] Gross J. J. Thompson S. C. (2007). Emotion regulation: conceptual foundations, in The Handbook of Emotion Regulation, ed Gross J. J. (New York, NY: Guilford Press), 3–24.

[B30] Gullone E. Hughes E. K. King N. J. Tonge B. (2010). The normative development of emotion regulation strategy use in children and adolescents: a two year follow-up study. J. Child Psychol. Psychiatry 51, 567–574. 10.1111/j.1469-7610.2009.02183.x 19845818

[B31] Gullone E. Taffe J. (2012). The emotion regulation questionnaire for children and adolescents (ERQ–CA): a psychometric evaluation. Psychol. Assess. 24, 409–417. 10.1037/a0025777 22023559

[B32] Hu L. Bentler P. (1999). Cutoff criteria for fit indexes in covariance structure analysis: conventional criteria versus new alternatives. Struct. Eqn. Model. 6, 1–55. 10.1080/10705519909540118

[B33] Ioannidis C. A. Siegling A. B. (2015). Criterion and incremental validity of the emotion regulation questionnaire. Front. Psychol. 6, 1–10. 10.3389/fpsyg.2015.00247 25814967 PMC4356000

[B34] Jazaieri H. Morrison A. S. Goldin P. R. Gross J. J. (2015). The role of emotion and emotion regulation in social anxiety disorder. Curr. Psychiatry Rep. 17, 1–9. 10.1007/s11920-014-0531-3 25413637

[B35] John O. P. Gross J. J. (2004). Healthy and unhealthy emotion regulation: personality processes, individual differences, and lifespan development. J. Pers. 72, 1301–1333. 10.1111/j.1467-6494.2004.00298.x 15509284

[B36] LaFontana K. Cillessen A. H. N. (2009). Developmental canges in the priority of perceived status in childhood and adolescence. Soc. Dev. 19, 130–147. 10.1111/j.1467-9507.2008.00522.x

[B37] La Greca A. M. López N. (1998). Social anxiety among adolescents: linkages with peer relations and friendships. J. Abnorm. Child Psychol. 26, 83–94. 10.1023/A:1022684520514 9634131

[B38] Lorenzo-Seva U. Ferrando P. J. (2006). FACTOR: a computer program to fit the exploratory factor analysis model. Behav. Res. Methods Instrum. Comput. 38, 88–91. 10.3758/BF03192753 16817517

[B39] Matsumoto D. Yoo S. H. Nakagawa S. Alexandre J. Altarriba J. Anguas-Wong A. M.. (2008). Culture, emotion regulation, and adjustment. J. Pers. Soc. Psychol. 94, 925–937. 10.1037/0022-3514.94.6.925 18505309

[B40] Melka S. E. Lancaster S. L. Bryant A. R. Rodríguez B. F. (2011). Confirmatory factor and measurement invariance analyses of the emotion regulation questionnaire. J. Clin. Psychol. 67, 1283–1293. 10.1002/jclp.20836 21928369

[B41] Neukrug E. Fawcett R. (2014). Essentials of Testing and Assessment: A Practical Guide for Counselors, Social Workers, and Psychologists. Stamford, CA: Cengage Learning.

[B42] Nezlek J. B. Kuppens P. (2008). Regulating positive and negative emotions in daily life. J. Pers. 76, 561–580. 10.1111/j.1467-6494.2008.00496.x 18399953

[B43] Ochsner K. N. Gross J. J. (2008). Cognitive emotion regulation: insights from social cognitive and affective neuroscience. Curr. Dir. Psychol. Sci. 17, 153–158. 10.1111/j.1467-8721.2008.00566.x 25425765 PMC4241349

[B44] Olivares J. Ruiz J. Hidalgo M. D. García-López L. J. Rosa A. I. Piqueras J. A. (2005). Social Anxiety Scale for Adolescents (SAS-A): Psychometric properties in a Spanish-speaking population. Int. J. Clin. Health Psychol. 51, 85–97.

[B45] Opitz P. C. Lee I. A. Gross J. J. Urry H. L. (2014). Fluid cognitive ability is a resource for successful emotion regulation in older and younger adults. Front. Psychol. 5, 1–13. 10.3389/fpsyg.2014.00609 24987387 PMC4060296

[B46] Rosenberg M. (1965). Society and the Adolescent Self-Image. Princeton, NJ: Princeton University Press.

[B47] Sala M. N. Molina P. Abler B. Kessler H. Vanbrabant L. van de Schoot R. (2012). Measurement invariance of the emotion regulation questionnaire (ERQ). A cross-national validity study. Eur. J. Dev. Psychol. 9, 751–757. 10.1080/17405629.2012.690604

[B48] Smetana J. G. Campione-Barr N. Metzger A. (2006). Adolescent development in interpersonal and societal contexts. Annu. Rev. Psychol. 57, 255–284. 10.1146/annurev.psych.57.102904.190124 16318596

[B49] Southam-Gerow M. A. Kendall P. C. (2002). Emotion regulation and understanding: implications for child psychopathology and therapy. Clin. Psychol. Rev. 22, 189–222. 10.1016/S0272-7358(01)00087-3 11806019

[B50] Teixeira A. Silva E. Tavares D. Freire T. (2014). Portuguese validation of the emotion regulation questionnaire for children and adolescents (ERQ-CA): relations with self-esteem and life satisfaction. Child Indic. Res. 8, 605–621. 10.1007/s12187-014-9266-2

[B51] Werner K. H. Goldin P. R. Ball T. M. Heimberg R. G. Gross J. J. (2011). Assessing emotion regulation in social anxiety disorder: the emotion regulation interview. J. Psychopathol. Behav. Assess. 33, 346–354. 10.1007/s10862-011-9225-x

[B52] Westermann S. Boden M. T. Gross J. J. Lincoln T. M. (2013). Maladaptive cognitive emotion regulation prospectively predicts subclinical paranoia. Cognit. Ther. Res. 37, 881–885. 10.1007/s10608-013-9523-6

[B53] Woods S. A. Hampson S. E. (2005). Measuring the big five with single items using a bipolar response scale. Eur. J. Pers. 19, 373–390. 10.1002/per.542

[B54] Woodward S. H. Shurick A. A. Alvarez J. Kuo J. Nonyieva Y. Blechert J.. (2015). A psychophysiological investigation of emotion regulation in chronic severe posttraumatic stress disorder. Psychophysiology 52, 667–678. 10.1111/psyp.12392 25516381

